# Dietary Nutrient Deficiencies and Risk of Depression (Review Article 2018–2023)

**DOI:** 10.3390/nu15112433

**Published:** 2023-05-23

**Authors:** Magdalena Zielińska, Edyta Łuszczki, Katarzyna Dereń

**Affiliations:** Institute of Health Sciences, College of Medical Sciences, University of Rzeszow, 35-959 Rzeszow, Poland

**Keywords:** nutrients, depression, nutrition, mental health, mood disorders

## Abstract

Depression is classified as one of the most common mental disorders. Its prevalence has recently increased, becoming a growing public health threat. This review focuses on clarifying the role and importance of individual nutrients in the diet and the impact of nutrient deficiencies on the risk of depression. Deficiencies in nutrients such as protein, B vitamins, vitamin D, magnesium, zinc, selenium, iron, calcium, and omega-3 fatty acids have a significant impact on brain and nervous system function, which can affect the appearance of depressive symptoms. However, it is important to remember that diet in itself is not the only factor influencing the risk of or helping to treat depression. There are many other aspects, such as physical activity, sleep, stress management, and social support, that also play an important role in maintaining mental health. The data review observed that most of the available analyses are based on cross-sectional studies. Further studies, including prospective cohort, case-control studies, are recommended to draw more reliable conclusions.

## 1. Introduction

Depression is classified as one of the most common mental disorders [1]. Its prevalence has recently increased, becoming a growing public health threat [1,2,3]. In 2019, the incidence rate of mental disorders was one in eight people worldwide, a significant epidemiological and health proportion [4]. Furthermore, it is suggested that nearly half of the population (around 44%) will experience at least one episode of a mental disorder during their lifetime [5]. Depression can be described as a complex and multicausal disease that involves a number of determinants, for example, biological, genetic, social, psychological, that are interrelated and interact with each other [6]. The prevalence of depressive disorders is also related to age (it most often starts in people between 20 and 40 years of age), gender (women fall twice as often as men), marital status (separated, divorced, widowed, especially for men), but also often to difficult traumatic experiences, for example, from childhood [7,8]. Other disease entities also contribute to the risk of depression, with particularly high prevalence rates associated with metabolic disorders (for example, cardiovascular disease) and autoimmune disorders [9,10]. Currently, a growing number of researchers are seeking answers to the question of whether diet can be a modifiable factor in mental health [11,12]. There is some evidence to suggest that diet and related eating habits may be indirectly related to the risk of onset, severity, and duration of depression [12,13]. Diet and related eating habits have been identified as protective factors against psychiatric disorders, as there are similar biological mechanisms that link depression and cardiometabolic diseases. Based on these, a theory has been presented that a proper diet can improve mental health and prevent the occurrence of depression, as well as its effect on the risk of cardiovascular disease [14,15]. Potential relationships between nutritional deficiencies and mental health are suggested to have some relevance to overall society’s well-being on a global scale, although current evidence is severely limited. Considering their role in depression can provide additional clues to the mechanisms that affect this disease and potentially contribute to the development of primary and secondary prevention strategies. However, it should be noted that scientific evidence on this issue is currently severely limited [16]. There are several treatments for depression, such as pharmacotherapy, psychotherapy, and non-pharmacological somatic treatments; however, as Blackburn writes, the treatment of depression has been suboptimal for 50 years [17,18,19]. Given the delayed onset of therapeutic effects and the desire to minimize side effects, particularly in cases of treatment-resistant depression, there is a growing interest in exploring strategies to accelerate the action of antidepressants [20]. Therefore, it is necessary to explore modifiable risk factors and effective prevention methods for depression [20,21,22]. To our knowledge, reviews have recently been published on the use of dietary interventions for depression in adults [23,24]. In terms of studies assessing the relationship between nutrient intake and risk of depression, some results remain inconclusive, and a comprehensive synthesis of existing data in this area is currently lacking. Therefore, this review focuses on clarifying the role and importance of individual nutrients in the diet and the impact of nutrient deficiencies on the risk of depression.

## 2. Methodology

The literature on the impact of nutrient deficiencies on depression was reviewed over the last five years (between 2018 and 2023). Internet databases (PubMed, Science Direct, Web of Science, Scopus, and Google Scholar) were searched according to keywords related to depression and nutrition, i.e., “nutritional deficiencies”, “depression”, “vitamin B”, “vitamin D”, “omega-3”, “adult depression”, etc. During the literature analysis, a total of 45 articles were identified that were dedicated to investigating the relationship between the intake of dietary nutrients and the appearance of depressive symptoms. The articles found were described in dedicated chapters and presented in tabular form, allowing the results presented to be compared and made more understandable. Furthermore, in addition to the aforementioned analysis of the scientific literature, a description of the relevance of nutrients for the functioning of the nervous system was made, and previous data indicating their potential impact on the risk of depressive symptoms were presented. Table 1 shows the inclusion and exclusion criteria for the studies in the review.

## 3. Dietary Nutrient Deficiencies and Risk of Depression

Depression is a heterogeneous disorder that is associated with biological mechanisms such as inflammation, dysregulation of the hypothalamic–pituitary–adrenal axis, dysfunction of the sympathetic and parasympathetic nervous system, and endothelial dysfunction [25]. Neurobiological investigations have revealed that depression is linked to cortical and limbic neuronal atrophy, as well as disrupted connectivity and functioning of neural networks. These changes are the result of structural, functional, and neurochemical deficits, of which problems related to γ-aminobutyric acid (GABA) and glutamate function are particularly relevant [26]. One of the most common hypotheses regarding the pathophysiology of depression suggests that there is also a link between depression and low levels of monoamine neurotransmitters. Reduced levels of serotonin, dopamine, and norepinephrine are observed in depressed individuals [27,28,29]. Synthesis and release of the aforementioned neurotransmitters depend on a number of factors, including changes in plasma composition, which are influenced by, among other things, the presence of nutrients in the diet. In addition, dietary modifications, such as changes in macronutrient, vitamin, and mineral intake, can affect cognitive function by regulating inflammatory processes and influencing molecular systems and cellular processes in the body [30,31]. Consequently, the role of optimising the supply of nutrients in the diet, which are essential for proper brain function and can support monoamine-based drug therapy, is increasingly emphasised in depressive disorders [32,33].

### 3.1. Macronutrients and Water

There is a scarcity of research that examines the impact of insufficient macronutrient intake in the diet on the likelihood and manifestations of depression. In the context of evaluating the impact of diet on health, including mental health, it is worth considering not only individual macronutrients, but also their proportions and the general diet of an individual [34]. The type and quantity of dietary protein consumed play an important role in the pathophysiology of depressive disorders, as protein serves as a crucial source of amino acids that serve as precursors to neurotransmitters within the brain. These are synthesised from aromatic amino acids, such as serotonin from tryptophan, and norepinephrine from tyrosine or its precursor phenylalanine. Therefore, a deficiency of these amino acids in the diet can result in reduced levels [35]. A study conducted by Kofler et al. showed a significant decrease in the concentrations of the aforementioned amino acids in the brain of individuals exhibiting depressive behaviour [36]. Studies on the impact of fat intake on the risk of depression most often analyse the quality of fat consumed, that is, the composition of fatty acids, and not just the amount of total fat consumed. A diet rich in saturated fatty acids (SFAs), trans fatty acids, may increase the risk of depression, while a diet rich in monounsaturated fatty acids (MUFAs) and polyunsaturated fatty acids (PUFAs) may reduce the risk of depression, although research is inconclusive [37,38]. The brain uses carbohydrates as a major source of energy, as well as structural and functional components [39]. They can also affect mood and brain function by affecting levels of neurotransmitters such as serotonin, dopamine, and norepinephrine [25]. In addition, the availability of neurotransmitters such as glutamate, acetylcholine, and GABA is modulated by the exogenous glucose supply [40]. A diet based on carbohydrates with a lower glycaemic index can effectively reduce the risk of depression [41]. A significant effect of a high glycaemic load diet was also observed on the risk of depression [42]. It is also worth mentioning a very important part of the diet, fibre, and its potential impact on the prevention of depression [43]. Based on the findings of the present meta-analysis, each 5 g increase in total dietary fibre intake is associated with a 5% reduction in the risk of depression [44]. An inverse significant relationship was observed between the intake of fibre from vegetables and soluble fibre and the likelihood of depression. However, fibre from cereal products, fruit, and insoluble fibre were marginally associated with a reduction in depression. The relationship between dietary water intake and depression is not fully understood, and a cross-sectional study on this topic was found during the review period. Water accounts for 75% of brain mass, and dehydration can affect nervous system function [45]. According to Liska et al., anger, confusion, depression, and fatigue increase with dehydration at ≥1% [46]. The association of the intake of the described nutrients with depressive symptoms is described in Table 2.

### 3.2. Polyunsaturated Fatty Acids

PUFAs are polyunsaturated fatty acids that the human body cannot synthesise on its own and are provided in the diet or by supplementation [63]. Furthermore, they show pleiotropic effects on neuronal structure and function, have anti-inflammatory effects, modulate neuroendocrine pathways, and activate key neurotransmitters, which can contribute to the prevention of depression [64,65]. The bulk of the dry weight of the mature brain is made up of fatty acids, with PUFAs making up about 20% of the dry weight. Both omega-3 fatty acids, mainly docosahexaenoic acid (DHA) and eicosapentaenoic acid (EPA), and omega-6 fatty acids, mainly arachidonic acid (AA), can be found in the brain [66]. DHA acid plays a key role, as it is quantitatively the most important fatty acid and is essential for the proper functioning of the neuronal membrane [67]. Consumption of omega-3 PUFA at lower levels or lower serum levels of these acids are associated with a higher risk of suicide attempts and an episode of major depression [68]. Supplementation with omega-3 PUFA fatty acids has the potential to demonstrate favourable outcomes in the prevention and management of depressive disorders, although the findings across studies are not consistently conclusive [64,69]. Table 3 shows the scientific studies that evaluated the intake of PUFA in the diet and its effect on the risk of depression.

### 3.3. Vitamins

#### 3.3.1. Vitamins of the B Group

Vitamin deficiency can be associated with symptoms of mental disorders [76]. Vitamins of the B group, in addition to the range of functions they perform in the human body, are essential for maintaining the normal functioning of the nervous system through the production of monoamine oxidase, DNA synthesis and methylation, and repair and maintenance of phospholipids [77]. In particular, deficiencies in B vitamins, i.e., B1, B6, B9, and B12, have been linked to depression, as they are essential for neuronal function [78,79,80]. They also have a protective effect against hypercysteinaemia, associated with an increased risk of mood disorders [81]. Furthermore, low levels of vitamin B9 and B12 have been associated with a poorer response to antidepressants [82]. Current work by Berkins et al. suggests that dietary intake of vitamin B6 and B12 may have an impact on brain structure. Vegetarians, particularly those who experience depression, can derive benefits from supplementing their diet with vitamins B6, B9, and B12 to promote optimal brain health [83]. The effects of the listed B vitamins on the nervous system and the risk of depression are described in Table 4.

Table 5 shows the current literature on the assessment of the dietary intake of B vitamins in the diet and their impact on the risk of depression. Low levels of the vitamins described can be due to a number of reasons, i.e., comorbidities, for example, multiple sclerosis, in which, according to a paper by Orti et al., patients who consumed less thiamine showed higher levels of depression characteristic of multiple sclerosis [93], old age [94], use of plant-based diets [95], their malabsorption [96], or unbalanced diet, so given the purpose of this review and the inclusion and exclusion criteria, the focus was mainly on dietary deficiencies in adults.

#### 3.3.2. Vitamin D

Vitamin D is a fat-soluble vitamin that is obtained during exposure to sunlight, food, and supplementation. An increasing number of studies confirm its role in the pathophysiology of diseases, including mental illness [104,105]. According to Fipps et al., the risk of depression increases by 8–14% in people with vitamin D deficiency [106]. Several mechanisms can be invoked to account for this phenomenon. One is the presence of vitamin D receptors (VDR) in different parts of the cortex and limbic system [107]. Many of these areas of the brain have been associated with the pathophysiology of depression, and VDRs are also found in the hippocampus, which plays a role in the control of memory and emotional function [108]. Furthermore, vitamin D is a potent regulator of the production of neurotrophic substances, such as brain-derived neurotrophic factor (BDNF), neurotrophin (NT)-3, and nerve growth factor (NGF). Subsequently, vitamin D plays an important modulatory role in regulating immunoinflammatory pathways that are important in the pathophysiology of depression, and lower serum vitamin D levels have been shown in depressed patients compared to controls [109,110,111]. Another hypothesis is the high levels of reactive oxygen species and Ca^2+^ in nerve cells in individuals deficient in vitamin D [112]. In addition, vitamin D is effective in preventing depression by maintaining serotonin levels at optimal levels and regulating dopamine and norepinephrine levels in the brain [113,114]. Vitamin D deficiency in patients with depression, as well as in the healthy population, is an important health problem. Research focusses mainly on laboratory measurements of serum vitamin D-25-hydroxyvitamin D (25(OH)D) levels [115]. The role of vitamin D in preventing the onset of postnatal depression is particularly highlighted [116]. An interesting area of research in the context of vitamin D and depression is the determination of levels of the vitamin D-binding protein gene. Calcitriol (1,25[OH]2D3) exerts its influence on the quantity and structure of the neurone by means of detoxification pathways, which encompass the inhibition of inducible nitric oxide synthase and the elevation of glutathione levels, together with the regulation of neurotrophin synthesis—all of which represent determinants associated with the development of depressive symptoms [117]. The potential influence of calcitriol on depressive symptoms can be attributed to the existence of the vitamin D receptor, the vitamin D-binding protein (VDBP), and/or the enzyme 1-alpha-hydroxylase, responsible for the conversion of 25(OH)D3 to 1,25(OH)2D3 within the central nervous system [117]. Table 6 shows the results of the studies on dietary intake and serum vitamin D levels and their impact on the risk of depressive symptoms.

### 3.4. Mineral Components

To date, scientific investigations have focused mainly on elucidating the involvement of minerals, assessing dietary and blood serum deficiencies, in the pathogenesis of depression [119,120]. Several of these, such as magnesium, zinc, iron, copper, and selenium, have been identified as key in the regulation of cell function and neuromodulation, as well as antioxidant activity [119,121]. Studies have shown that insufficient magnesium levels can induce alterations in central nervous system functioning, specifically affecting glutamatergic transmission within the limbic system and the cerebral cortex. These regions of the brain are critical in the pathophysiology of depression cause [122]. According to Pickering et al., magnesium deficiency can affect the secretion of corticotropin-releasing hormone, which, in turn, leads to increased levels of adrenocorticotropic hormone (ACTH). This mechanism may be related to depression by affecting the regulation of the hypothalamic–pituitary–adrenal (HPA) axis [123]. Magnesium is also an antagonist of NMDA receptors (N-methyl-d-aspartate), and its deficiency can lead to its functional overactivity [124]. The NMDA receptor is associated with the flow of calcium ions into nerve cells, which is crucial for their proper functioning. Its excessive activation can lead to increased calcium ion flow, which, in turn, leads to neurotoxicity. The relationship between dietary magnesium intake and serum magnesium levels and the risk of depression is currently inconclusive. It should be noted that serum magnesium represents only ~1% of total magnesium in the body and does not fully reflect intracellular magnesium status.

Research suggests that changes in brain zinc levels are associated with the development of depression and that zinc supplementation may help treat the condition. Zinc supplementation has been shown to support the effects of antidepressants and improve depressive symptoms in some patients [125]. Another possible reason for the antidepressant effect of zinc could be its anti-inflammatory and antioxidant properties [126]. Furthermore, zinc is also an NMDA receptor antagonist [127]. In addition, zinc influences hormonal regulation, including cortisol levels, cellular immune response, neurogenesis, neuronal plasticity, and expression of BDNF in the hippocampus and cortex, and zinc ions regulate synaptic transmission or act as neurotransmitters [128]. Recent scientific investigations have emphasised the importance of zinc transporters (ZincTs) and zinc-sensing GPR39 receptors in the aetiology and therapeutic interventions related to depression [129]. Current evidence from observational studies and RCTs supports the potential benefits of zinc in reducing risk and alleviating depression [130].

According to Chadern, selenium intake is the most associated with depression of all dietary factors considered [131]. Selenium is an essential trace element that is required for the proper functioning of several selenoproteins, including those involved in the defence of antioxidants in the brain and nervous system. In addition, it helps protect cells from oxidative damage, which can lead to inflammation and disease, including neurological diseases [132]. Given its neuromodulatory role in brain function, researchers are increasingly testing the link between selenium and depression, but some of the results are contradictory [133]. 

In a recently published study by Hongrong et al., the prevalence of depressive symptoms was significantly higher in patients with elevated serum copper levels. Among serum levels of copper, selenium, and zinc, only an association was observed between depressive symptoms and copper levels [134]. These findings are supported by a meta-analysis by Ni et al. suggesting that elevated blood copper levels may be associated with depressive disorders and, therefore, a possible role for copper as a biomarker of depression [135]. Although it can be speculated that the imbalance of copper in the human body may be one of the causes of depression, there are processes in which copper plays an important role in the development and function of the nervous system. These include neurogenesis, synaptogenesis, neurotransmission, cognition, learning and memory processes, and NMDA function [136]. Copper can support the activity of two neurotrophic factors: BDNF and nerve growth factor (NGF). These neurotrophic factors can influence neuronal plasticity and the neural network [137,138]. Taking a monoaminergic approach to depression, the role of copper in the conversion of dopamine to norepinephrine should also be mentioned. The conversion of dopamine to norepinephrine is copper-dependent, as copper ions interact with dopamine β-hydroxylase [139]. Copper deficiency and excess copper can affect brain function. Astrocytes are considered important regulators of the homeostasis of this element [140].

Iron deficiency represents a prevalent nutritional problem on a global scale and can cause iron deficiency anemia, which is one of the most frequently encountered forms of anemia. In particular, there has been a growing focus on investigating iron metabolism and its pivotal role in the context of mental disorders [141]. Norepinephrine has been linked to iron metabolism in the brain, which can affect the neuroplasticity and function of prefrontal neurones and the hippocampus. Furthermore, BDNF levels are regulated by iron, which is necessary for normal synthesis of neurotransmitters and also for the aromatic hydroxylase enzymes present in neurones. For this reason, the neurobioavailability of iron and the ability of the brain to capture iron from the blood are important for maintaining adequate levels of BDNF and neurotransmitters involved in emotional processes, including depression [142]. The hypothesis that dysfunctions in the glutamatergic system may be associated with the appearance of depression is currently under intensive investigation and is not yet fully confirmed. However, there is evidence that iron deficiency can affect changes in the glutamatergic system and lead to mood disorders. Iron plays an important role as an enzyme cofactor in metabolic reactions that lead to the production and release of the neurotransmitter glutamate [143]. A recent review by Wassef et al. found that anemia and/or iron deficiency may contribute to postpartum depression in women at risk [144]. Richardson et al. showed that higher body iron levels in young adult males may be associated with more depressive symptoms [145].

The role of calcium in the pathophysiology of depression can be explained by its involvement in several mechanisms. One of these is the involvement in the regulation of the hypothalamic–pituitary–adrenal (HPA) system. Furthermore, extracellular calcium influx is an important component of many neuronal processes. Changes in extracellular Ca^2+^ concentration may be involved in emotion regulation, which may be a direct effect of Ca on stabilisation of the plasma membrane, and NMDA may also affect neuronal plasticity [146]. Table 7 shows, based on the current literature, studies that determined dietary elemental intake and self-reported depression.

## 4. Limitations

The article delineates the role and significance of nutritional components in the diet within the context of their impact on the nervous system and depressive symptoms. Furthermore, it presents research pertaining to the relationship between the consumption of nutritional components in the diet and their influence on depression. Nevertheless, it has some limitations. One is that it is mainly based on cross-sectional studies, which make it impossible to determine causal conclusions and need to be supplemented and confirmed by longitudinal clinical-control studies. However, it should be noted that observational studies are also a valuable source of information, contributing to the evaluation of the available scientific evidence. The studies included in this review were primarily based on observation of large groups of subjects, allowing more representative and general data. Furthermore, the diagnosis of depression was mainly based on self-report questionnaires, which assess depressive symptoms, but should not be considered equivalent to a clinical diagnosis of depression and therefore would need to be completed according to the DSM criteria. Another limitation is the evaluation of dietary nutrient intake, which was mostly done on the basis of food frequency questionnaires or 24 h interviews, which poses the risk of under or overestimation of data, although in most studies participants were carefully instructed on how to complete the questionnaire or dietary interview (for example, it was conducted twice, one in the presence of the research team person and the other a few days after, by telephone). However, it should be stressed that there is no perfect tool for assessing food intake and that, in most low-cost and simple methods, some limitations are inevitable. Furthermore, when nutrients are consumed together rather than separately, they can influence each other through their interactive or synergistic effects. Therefore, the study of dietary patterns is a second interesting area of research in addition to assessing the impact of individual nutrients.

## 5. Strengths and Future Research Directions

Given the current state of knowledge and the often contradictory results of studies dedicated to the impact of nutrition on mental health, this review seems to be a needed addition to this information. The number of articles included in this review can be seen as a strength. It is not easy to discuss the effectiveness of introducing nutritional interventions among people with a risk of developing or current psychiatric disorders. It can certainly be argued that the presented data show that nutrition should not be considered as the only means of preventing depression or replacing pharmacology. Additionally, dietary modifications appear to be an underused tool for improving mental health. The development of psychological nutrition faces many obstacles, including the difficulty of maintaining a healthy, varied diet, given the popularity of the Western diet and the difficulty of adhering to dietary recommendations among patients with depression.

This review also aims to spread awareness of nutrition and its impact on mental health and highlight it for further research. Risk groups such as pregnant women, in whom the need for many nutrients increases and the supply of these nutrients from the diet is insufficient, are also very important [161]. The nutrients ingested by pregnant women are essential to facilitate normal growth and development of the foetus, particularly with respect to the maturation of the nervous system [162]. It is recommended that more work should be done to create clear dietary recommendations for the risk and prevalence of mental health problems. This aspect should also be taken into account in supplementation programmes.

It is also worth noting that behavioural factors such as physical inactivity, excessive alcohol consumption and smoking, and sleep deprivation can influence poor eating habits and lead to nutrient deficiencies [163]. For example, people with sleep disorders often have a greater tendency to eat highly processed and nutritionally poor foods, which can lead to nutritional deficiencies [163,164]. In addition, overweight and obesity, often as a consequence of unhealthy lifestyles, are linked to depressive disorders [165]. Alcohol and tobacco can also affect nutrient absorption and metabolism, contributing to deficiencies. These deficiencies can further affect neurochemical balance and brain function, contributing to the development or exacerbation of depressive symptoms [166]. It is worth considering these factors in future research into the role of nutrition in depression and to approach healthy lifestyles in a holistic way, taking into account the various aspects that support mental health [167,168,169,170]. Research that considers these aspects may provide a more comprehensive understanding of the mechanisms through which these factors influence the development and course of depression. This information may be valuable for the development of more effective dietary strategies and interventions to alleviate the symptoms of depression by optimising nutrients and changing unhealthy lifestyle behaviours.

## 6. Conclusions

Deficiencies in nutrients such as protein, B vitamins, vitamin D, magnesium, zinc, selenium, iron, calcium, and omega-3 fatty acids have a significant impact on the function of the brain and nervous system, which can affect the appearance of depressive symptoms. On the other hand, an excess of certain nutrients in the diet can also have a negative impact on mental health (copper, iron). To prevent deficiencies of the listed nutrients in the diet, it is worth noting the importance of a varied and balanced diet, while also taking into account the potential risks associated with the absorption and bioavailability of certain components through the presence of other components in the diet [171]. In summary, based on the available scientific evidence, a diet that supports mental health should consist mainly of vegetables, fruits, whole grains, sources of vegetable protein, legumes, nuts, fish, low-fat dairy products, while limiting the intake of simple sugars and highly processed foods [172]. The indicated food sources contain vitamins, minerals, tryptophan, fibre, antioxidants, and good quality fats, which can benefit mental health by reducing oxidative stress, inflammation and providing essential nutrients for the brain. However, it is important to remember that diet in itself is not the only factor influencing the risk of or helping to treat depression. There are many other aspects, such as physical activity, sleep, stress management, and social support, that also play an important role in maintaining mental health. It is worth approaching a healthy lifestyle in a comprehensive way, taking into account different aspects to support mental health. Further studies, including prospective cohort, case-control studies, are recommended to draw more reliable conclusions. Table 8 summarizes the effects of the nutrients described on the risk of depressive symptoms.

## Figures and Tables

**Table 1:** Eligibility criteria.

Inclusion Criteria	Exclusion Criteria
- Studies involving adults (people aged >18 years). - Observational studies (including cohort, longitudinal, and cross-sectional studies) and interventional studies (randomised controlled clinical trials). - Studies that focused on assessing dietary nutrient deficiencies. - The main focus was on major depressive disorder (MDD). - Papers were included regardless of the measurement methods used in the study (mainly self-reporting tools or medical diagnosis). - Articles published in English. - Studies published in the last five years (between 2018 and 2023).	- Animal studies, reviews, case reports, editorials. - Studies assessing depression as a secondary disease to another disorder and cancer. - Studies that assess depression in the context of nutrition after bariatric surgery. - Research only in paediatric and elderly populations and pregnant and/or breastfeeding women, as these are populations that would require a separate, stand-alone study on the topic described. - Studies that focused exclusively on evaluating the effectiveness of dietary interventions (for example, Western diet, Mediterranean diet, and others) or exclusively on nutrient supplementation. - Research that exclusively focusses on bipolar disorder. - Studies exclusively on depression and the gut–brain axis. - Articles published in a language other than English. - Studies published before 2018.

**Table 2:** Relationship between dietary macronutrients and water intake and the risk of depressive symptoms.

Authors	Year of Publication	Type of Study	The Group	Assessment of Depression and Dietary Intake	Results and Conclusions
**Proteins**					
Li et al. [47]	2020	National cross-sectional survey	n = 17,845 adults	Patient Health Questionnaire-9 (PHQ-9) and two 24 h dietary recall interviews	Total protein intake and protein intake from milk and dairy products may reduce the risk of depressive symptoms in American adults, but not for protein intake from red meat, poultry, fish, cereals, and legumes.
Sheikhi et al. [48]	2023	Cross-sectional study	n = 489 Iranian women	Depression, Anxiety, and Stress Scales (DASS) questionnaire and Semi-quantitative Food Frequency Questionnaire	Women were more likely to show symptoms of depression in the highest tertile of animal protein intake. A diet high in animal protein may predispose to mental illness.
**Amino acids**					
Suga et al. [49]	2018	Cross-sectional study	n = 7923 adults	Center for Epidemiologic Studies Depression Scale (CES-D) and validated, self-administered diet history questionnaire	Women with depression consumed more energy and less tryptophan than women without depression. Tryptophan intake was inversely associated with depressive symptoms in young adult women.
Koochakpoor et al. [50]	2021	Cross-sectional study	n = 3175 adults	Hospital Anxiety and Depression Scale (HADS) and Food Frequency Questionnaire (FFQ)	Subjects in the study with the highest total branched-chain amino acid (BCAA) tercile intake had a lower risk of depression. There was also a significant inverse relationship observed between isoleucine intake and the likelihood of depression.
Reuter et al. [51]	2021	Cross-sectional study	n = 482 adults	Beck Depression Inventory-II (BDI-II) and participants were asked for their general consumption of TRP-rich foods	A diet rich in tryptophan has been shown to have a positive effect on mood. High amounts of tryptophan in the diet appear to protect against depression and have a positive effect on social interactions.
**Fats**					
Wilson et al. [52]	2021	Cross-sectional study	n = 887 adults	Beck Depression Inventory-II (BDI-II) and Dietary Instrument for Nutrition Education questionnaire (DINE)	No relationship was observed between the amount of fat ingested in the diet and mental health.
Currenti et al. [53]	2023	Cross-sectional study	n = 1572 adults	Center for the Epidemiological Studies of Depression Short Form (CES-D and two Food Frequency Questionnaires (FFQs; a long and a short version)	No association was found between total fat intake and depressive symptoms.
**Carbohydrates**					
Sanchez-Villegas et al. [54]	2018	Prospective cohort study	n = 15,546 adults	Follow-up questionnaire and diagnosis of depression using a clinical interview for DSM-IV and Semi-quantitative FFQ	Greater exposure to added sugars and low-quality carbohydrates correlate with a higher risk of depression.
Ebrahimpour-Koujan et al. [55]	2019	Cross-sectional study	n = 3362 adults	Hospital Anxiety and Depression Scale and General Health Questionnaire-12 and Semi-quantitative Food Frequency Questionnaire	Depression was not associated with adherence to a low-carbohydrate diet.
Makhani et al. [56]	2021	Cross-sectional study	n = 9728 adults	Patient Health Questionnaire-9 (PHQ-9) and two 24 h dietary recall interviews	The higher the ratio of carbohydrate-to-fibre intake in the diet, the higher the risk of depressive symptoms, which can be moderate to severe in severity.
Amirinejad et al. [57]	2022	Cross-sectional study	n = 7384 adults	Depression Anxiety Stress Scales and Validated Food Frequency Questionnaire	No significant association was observed between the dietary glycaemic index (DGI) and glycaemic load (DGL) and the likelihood of depression.
**Total macronutrients**					
Pooyan et al. [58]	2018	Cross-sectional study	n = 265 adults	Depression Anxiety Stress Scales 21 (DASS-21) and Semi-quantitative Food Frequency Questionnaire and blood samples were taken, and biochemical measurements were taken	A significant interaction was found between the high-protein and low-fat diets and the low-fat diet and the rs7041 polymorphism in groups with moderate and severe depression. In healthy adults without chronic diseases, a high-protein, low-fat diet may interact with the VDBP genotype to reduce the risk of depression.
Oh et al. [59]	2020	Cross-sectional study	n = 76,635 (60,935 from the United States and 15,700 from South Korea)	Patient Health Questionnaire-9 (PHQ-9) and 24 h dietary recall interview	In both the United States and South Korea, people with low protein intake had a significantly higher risk of depression than those with normal protein intake. When the proportion of kilocalories consumed from protein increased by 10%, the prevalence of depression was significantly reduced. There was no significant association between fat intake and depression in either country. An association was observed between carbohydrate intake and the incidence of depression in the United States, but not in South Korea.
Eissenstat et al. [60]	2020	Cross-sectional study	n = 4747 adults	Patient Health Questionnaire-9 (PHQ-9) and two 24 h dietary recall interviews	For respondents of Hispanic origin, total protein intake and fats were negatively associated with depressive symptoms. In Caucasians, the intake of dietary fibre was negatively associated with symptoms of depression.
Lee et al. [61]	2021	Cross-sectional study	n = 6336 adults (3102 Korean adults and 3234 Americans)	Patient Health Questionnaire-9 (PHQ-9) and 24 h dietary recall interview	A low intake of protein and dietary fibre as a result of food insecurity can increase the risk of depression. Adequate diet and food security can play an important role in the prevention of depression.
**Water**					
Haghighatdoost et al. [62]	2018	Cross-sectional study	n = 3327 adults	Hospital Anxiety and Depression Scale and water consumption was assessed by asking about the number of glasses of water that were consumed daily (<2, 2–5, and ≥5 glasses of water/day)	A significant inverse relationship was shown between water consumption and depression. Drinking <2 glasses of water per day was associated with a 73% increased risk of depression in men and 54% in women.

**Table 3:** Assessment of dietary fatty acid intake and prevalence of depressive symptoms.

Authors	Year of Publication	Type of Study	The Group	Assessment of Depression and Dietary Intake	Results and Conclusions
Sánchez-Villegas et al. [70]	2018	Cross-sectional study	n = 6874 adults	Beck Depression Inventory-II and 143-item Semi-quantitative Food-Frequency Questionnaire	Total omega-3 fatty acid intake (approximately 0.5–1 g/day) was significantly associated with a lower incidence of depression.
Park et al. [71]	2020	Cohort study	n = 2200 adult women	Beck Depression Inventory-II (BDI-II) and Center for Epidemiologic Studies-Depression scale (CES-D) assessment questionnaires, and n-3 FA intakes were assessed using a Semi-quantitative Food-Frequency Questionnaire	A higher intake of omega-3 fatty acids was associated with increased connectivity within the neural networks associated with emotion and attention, and increased connectivity between the regions of the brain associated with emotion control and cognitive processes. Omega-3 fatty acid intake may have an effect on brain function and possibly a role in the reduction of depressive symptoms in middle-aged women.
Zhang et al. [72]	2020	Cross-sectional study	n = 17,431 adults	PHQ-9 (nine-item Patient Health Questionnaire) and two 24 h dietary recall interviews	A higher intake of omega-3 fatty acids was associated with a lower risk of depressive symptoms, while a higher intake of omega-6 fatty acids was associated with a higher risk of depressive symptoms. A higher omega-6:omega-3 ratio was also associated with a higher risk of depressive symptoms.
Li et al. [73]	2020	Cross-sectional study	n = 3054 adult women	Center for Epidemiological Studies Depression Scale (CES-D) and Food Frequency Questionnaire (FFQ)	A higher intake of omega-3 fatty acids was associated with a lower risk of depression in early perimenopausal women.
Berger et al. [74]	2020	Cross-sectional study	n = 206 adults	Patient Health Questionnaire-9 (PHQ-9) and diet with a structured questionnaire	Higher intakes of seafood and omega-3 fatty acids were associated with a lower risk of depressive symptoms. A protective factor against depression may be the availability of fresh seafood in the local diet.
Chaves et al. [75]	2022	Longitudinal study	n = 13,879 adults	The Clinical Interview Schedule Revised (CIS-R) and Food Frequency Questionnaire (FFQ)	The protective effect of omega-3s (total and subtypes) has been shown, with reductions of 2–65% in the risk of major depressive disorder. Consumption of all types of omega-3 fatty acids was lower among those with persistent depressive episodes.

**Table 4:** Main role of selected B vitamins in the nervous system.

Vitamin B	Effects on the Nervous System and Risk of Depression
**Vitamin B1** **(thiamine)**	Thiamine is an important coenzyme during the synthesis of neurotransmitters, such as acetylcholine and serotonin, for example. The most important function of thiamine is considered to be that it makes a major contribution to cellular energy metabolism and, as an essential cofactor in carbohydrate metabolism, it helps to supply energy to nerve cells [84]. An inverse relationship has been shown between thiamine levels and depressive symptoms in adults [85].
**Vitamin B6** **(pyridoxine)**	Pyridoxine functions as a cofactor in the pathways involved in myelin synthesis and enzymatic reactions, including the synthesis of neurotransmitters such as gamma-aminobutyric acid (GABA), serotonin, and dopamine [86]. Furthermore, it controls glutamate excitability and neuronal metabolism. Vitamin B6 and magnesium both modulate neurobiological mechanisms, leading to speculation that they may exert synergistic effects [87]. Pyridoxal-5-phosphate concentrations, the active form of vitamin B6, were measured in Hispanic adults in years 2 and 5 of the study, and it was observed that depressive symptoms were higher in those with low values [88].
**Vitamin B9** **(folic acid)**	Folic acid is involved in the synthesis and metabolism of neurotransmitters associated with depression (serotonin, dopamine, norepinephrine). In addition, it plays a vital role in the regeneration of tetrahydrobiopterin (BH4), a cofactor essential for the formation of neurotransmitters [89]. An association has been shown between lower serum folic acid levels during pregnancy and prenatal depression [90].
**Vitamin B12** **(cobalamin)**	A specific function of vitamin B12 is to participate in the DNA synthesis of myelin-producing oligodendrocytes and in the synthesis of myelin [83,85]. Cobalamin is a cofactor of the methionine synthase enzyme, which catalyses the reaction to transfer a methyl group to a homocysteine molecule. Methionine is formed, which is the precursor of S-adenosylmethionine (SAM). SAM plays an important role in the methylation processes necessary for the normal synthesis and/or metabolism of membrane phospholipids, DNA, RNA, neurotransmitters and for the normal function of the myelin sheaths of nerve fibres [91]. Vitamin B12 deficiency may be associated with impaired glutathione peroxidase activity elevated levels of free radicals. Furthermore, the prevalence of depression tends to be higher among vegetarians due to insufficient intake of vitamin B12 [92].

**Table 5:** Assessment of dietary intake of B vitamins and prevalence of depressive symptoms.

Authors	Year of Publication	Type of Study	The Group	Assessment of Depression and Dietary Intake	Results and Conclusions
**Vitamin B1**					
Duc et al. [97]	2021	National cross-sectional survey	n = 34,700 adults	Depression was defined as physician diagnosis, the current presence or treatment for depression and 24 h dietary recall interview and a Semi-quantitative Questionnaire on Food Frequency	Serum vitamin B1 concentrations were assessed and related to dietary intake. Low serum thiamine concentrations were associated with a high incidence of depressive symptoms. Increased daily vitamin B1 intake was negatively associated with the appearance of depression.
Nguyen et al. [98]	2022	National cross-sectional survey	n = 16,371 adults	Depression was defined based on the diagnosis of the physician or the current presence or treatment of depression, and daily food intake was calculated using the 24 h recall method and a Semi-quantitative Food Frequency Questionnaire (FFQ)	A higher dietary intake of vitamin B1, B3, or A reduces the risk of depression in a nationally representative Korean cohort.
**Vitamin B6**					
Kafeshani et al. [99]	2020	Cross-sectional study	n = 3362 adults	Hospital Anxiety and Depression Scale (HADS) and a validated, 106-item Food Frequency Questionnaire (FFQ)	The average intake of vitamin B6 (mg/day) was lower in people with depression. A lower intake of vitamin B6 in the general population and among women was associated with a higher likelihood of depression.
Odai et al. [100]	2020	Cross-sectional study	n = 289 adult women	Hospital Anxiety and Depression Scale (HADS) and self-administered diet history questionnaire (BDHQ)	Symptoms of moderate-to-severe depression were associated with a lower dietary intake of vitamin B6. Symptoms of depression may be reduced by increasing your intake of vitamin B6.
**Vitamins B9 and B12**					
Khosravi et al. [101]	2020	Clinical-control study	n = 260 women (87 depressed and 173 healthy) and 70 men (22 depressed and 48 healthy)	The major depressive disorder(s) of the participants was diagnosed by psychiatrists using the criteria of DSM-IV and for the control group Beck Depression Inventory questionnaire (BDI-II), and the dietary intake in the last 12 months was evaluated using a Semi-quantitative Food Frequency Questionnaire (FFQ).	In addition to dietary nutrient intake, their serum concentrations were evaluated and related to each other. An unhealthy diet was associated with a higher risk of depression through a reduction in the serum levels of vitamins B9 and B12.
Zheng et al. [102]	2020	Cross-sectional study	n = 19,244 adults	Patient Health Questionnaire-9 (PHQ-9) and 24 h dietary recall interview	Synthetic folic acid was not associated with depressive symptoms, but total and natural folate intakes were inversely associated with depressive symptoms.
Mahdavifar et al. [103]	2021	A population-based prospective cohort study	n = 7387 Iranian adults	Iranian validated version of depression, anxiety, and stress scale questionnaire 21 (DASS 21) and Semi-quantitative Food Frequency Questionnaire (DS-FFQ)	Low intakes of B vitamins, including folic acid, vitamin B6, and vitamin B12, were associated with higher depressive symptoms. Higher biotin intake was significantly associated with lower incidence of depressive symptoms.

**Table 6:** Assessment of dietary intake and serum vitamin D levels and prevalence of depressive symptoms.

Authors	Year of Publication	Type of Study	The Group	Assessment of Depression and Dietary Intake	Results and Conclusions
**Vitamin D**					
Pooyan et al. [58]	2018	Cross-sectional study	n = 265 adults	Depression Anxiety Stress Scales 21 (DASS-21) and Semi-quantitative Food Frequency Questionnaire (FFQ)	In healthy adults without chronic disease, a high-protein/low-fat diet may interact with the genotype of vitamin D-binding protein VDBP to reduce the risk of depression.
Jahrami et al. [118]	2020	Clinical-control study	n = 192 participants: 96 patients with depression and 96 age- and sex-matched controls	Beck Depression Inventory-II (BDI-II) and Food Frequency Questionnaire (FFQ) (covering 102 foods distributed on 38 items/groups)	Depressed patients have significantly lower serum vitamin D levels. Depressed patients appeared to have statistically significantly less vitamin D from sunlight sources, even though dietary vitamin D intake was the same in both groups. Approximately 80% of depressed patients and 70% of controls have been shown not to take adequate daily doses of vitamin D.

**Table 7:** Assessment of dietary mineral intake and prevalence of depressive symptoms.

Authors	Year of Publication	Type of Study	The Group	Assessment of Depression and Dietary Intake	Results and Conclusions
**Magnesium**					
Anjom-Shoae et al. [147]	2018	Cross-sectional study	n = 3172 adults	Hospital Anxiety and Depression Scale (HADS) and dish-based 106-item Semi-quantitative FFQ (DS-FFQ)	Among normal-weight men and overweight women, a significant inverse association was found between magnesium intake and depression.
Sun et al. [148]	2019	Cross-sectional study	n = 17,730 adults from the National Health and Nutrition Examination Survey	Patient Health Questionnaire (PHQ-9) and two 24 h dietary recall interviews	Lower magnesium intake was associated with a higher risk of depression, particularly in the female group. In all age groups, the inverse association between dietary magnesium intake and risk of depression was statistically significant.
Chou et al. [149]	2023	Cross-sectional study	n = 4615 adults	5-Item Brief Symptom Rating Scale (BSRS-5) and Serum Magnesium (mg/dL) and dietary magnesium intake (mg)—24 h dietary recall questionnaire	Serum magnesium concentration was inversely correlated with the occurrence of depressive symptoms, which was not shown for the dietary magnesium intake. Serum magnesium was poorly correlated with dietary magnesium. The level of serum magnesium was negatively associated with depressive symptoms in the sample, in the men, but not in the women.
**Zinc**					
Hajianfar et al. [150]	2021	Cross-sectional study	n = 142 female students	The Beck Depression Inventory-II (BDI-II) and Semi-quantitative Food Frequency Questionnaire (FFQ)	In Iranian female students, an inverse association was observed between dietary zinc intake and mood disorders, including depression, and some indicators of sleep disturbance.
Hu et al. [151]	2022	Cross-sectional study	n = 31,839 adults	Patient Health Questionnaire-9 (PHQ-9 and two 24 h dietary recall interviews	Especially in the female population, low dietary intakes of zinc and vitamin A were significantly associated with depression. In the low zinc intake group, the risk of depression was significantly reduced with increased total vitamin A intake.
**Selenium**					
Ghimire et al. [152]	2019	Cross-sectional study	n = 7725 adult participants in the National Health and Nutrition Examination Survey (NHANES)	Patient Health Questionnaire-9 (PHQ-9) and 24 h dietary recall interview	An inverse relationship was observed between participants’ dietary selenium intake and depressive symptoms. There was no association between serum selenium levels and depressive symptoms.
Ferreira de Almeida et al. [153]	2021	Cross-sectional study	n = 736 adult farmers	Mini-International Neuropsychiatric Interview and three 24 h dietary recall interviews	A high intake of selenium is associated with a lower incidence of depression, even after taking into account sociodemographic variables, lifestyle factors, and pesticide poisoning.
**Copper**					
Nakamura et al. [154]	2019	Cross-sectional study	n = 2089 adults	Kessler’s six-item psychological distress scale (K6) and the FFQ include 87 food items and ask about the usual consumption rates and portion sizes during the previous month.	Low intakes of zinc, copper, and manganese were associated with depressive symptoms. For calcium, magnesium, and iron, the inverse relationship was not statistically significant.
**Iron**					
Li et al. [155]	2018	Cross-sectional study	n = 14,834 adults	Patient Health Questionnaire (PHQ-9) and 24 h dietary recall	Participants with depression had significantly lower total daily zinc, iron, copper, selenium, and energy intakes than those without depression. Higher zinc, iron, copper, and selenium intake was negatively associated with depression, and negative associations with copper and selenium intake remained statistically significant after considering potential confounders.
**Calcium**					
Shen et al. [156]	2023	Cross-sectional study	n = 14,971 adults	Patient Health Questionnaire (PHQ-9) and 24 h dietary recall	Even after adjusting for a large number of potential confounders, calcium intake was negatively associated with the risk of depressive symptoms. The incidence of depressive symptoms decreased as calcium intake increased.
**Various Minerals**					
Sánchez-Villegas et al. [157]	2018	Cohort study	n = 13,983 students	136-item validated Semi-quantitative Food Frequency Questionnaire (FFQ) and structured clinical interview for DSM-IV (SCID-I)	A deficiency of 4 nutrients at baseline increased the risk of depression (folic acid, magnesium, calcium, and potassium) in this longitudinal study of a middle-aged population.
Salehi-Abargouei et al. [158]	2019	Cross-sectional study	n = 3846 adults	Hospital Anxiety and Depression Scale (HADS) and Semi-quantitative Food Frequency Questionnaire (FFQ)	It has been shown that higher intakes of B12, zinc, phosphorus, saturated fats, cholesterol, B5, and B6 are associated with a reduced risk of major depression in men and a reduced risk of psychological distress in women.
Yun et al. [159]	2021	Cross-sectional study	n = 10,106 adults	Patient Health Questionnaire-9 (PHQ-9) and nutrient intake and dietary habit information of the K-NHANES were used as independent variables.	Sugar, sodium, vitamin A, water, fat, saturated fatty acids, omega-6 fatty acids, dietary fibre, and frequency of breakfast, lunch, dinner, and eating out were significantly associated with depression.
Ferriani et al. [160]	2022	Longitudinal study	n = 14,737 adults	Clinical Interview Schedule Revised (CIS-R) and Food Frequency Questionnaire (FFQ)	A significant inverse relationship was found between depression and a higher intake of selenium, zinc, vitamins B6 and B12 in the sample. Among women, a similar pattern of correlation was observed, in addition to higher intakes of vitamins A and C. Among men, a significant inverse relationship between depression was only observed with vitamin B12 and B6 intake. The incidence and severity of depression may be reduced by increasing intake of selected micronutrients.

**Table 8:** Summary of the effects of the described nutrients on the risk of depression.

Nutrient	Conclusions
**Proteins**	Higher total protein consumption as well as that specifically derived from milk and dairy products might potentially lower the susceptibility to depressive symptoms. No benefit has been shown for protein intake from animal sources [47,48]. A beneficial effect of a higher supply of tryptophan in the diet has been shown to decrease the risk of depression [49,50,51]. A diet with an increased supply of protein relative to other macronutrients may have a beneficial effect on the risk of depression in adults who do not have any chronic diseases [58,59,60,61].
**Fats**	No association has been observed between total fat consumption and the manifestation of depressive symptoms [52,53,59], although not all results are conclusive [58,60].
**Carbohydrates**	Greater exposure to added sugars, low-quality carbohydrates, and low dietary fibre intake have been shown to correlate with a higher risk of depression [54,57]. No significant association was observed between the dietary glycaemic index and the dietary glycaemic load and the likelihood of depression [57].
**Water**	To date, it has not been conclusively demonstrated that dietary water intake directly affects the risk of depression; however, dehydration can exacerbate, for example, fatigue and depression. Therefore, it is worth ensuring that the body is adequately hydrated to avoid the negative effects of dehydration [46,62].
**Polyunsaturated fatty acids**	All the publications cited showed a beneficial effect of increased omega-3 fatty acid intake to prevent the onset of depressive symptoms in different groups [70,71,72,73,74,75].
**Vitamins of the B group**	Low dietary and/or serum levels of vitamins B1, B6, B9, and B12 have been associated with a higher prevalence of depressive symptoms [97,98,99,100,101,102,103]. The findings suggest that the intake of adequate amounts of B vitamins may have a positive impact on mental health and reduce the risk of depressive symptoms.
**Vitamin D**	Depressed patients have significantly lower serum vitamin D levels [58,118]. It has been shown that approximately 80% of depressed patients do not take adequate daily doses of vitamin D [118].
**Mineral Components**	Dietary deficiencies in magnesium, zinc, selenium, copper, and manganese have been associated with a greater likelihood of depression [147,148,149,150,151,152,153,154,155,156,157,158,159,160]. Both excess and deficiency of copper and iron may affect the risk of depression [135,145,154,155].

## Data Availability

Not applicable.
